# Supplementary material for: Empirical methods for controlling false positives and estimating confidence in ChIP-Seq peaks
Source: BMC Bioinformatics. 2008 Dec 5;9:523. doi: 10.1186/1471-2105-9-523 (PMC2628906; doi:10.1186/1471-2105-9-523)
Supplement: Additional File 1 — A variety of html documents from the USeq web site detailing the available applications, their best usage, output file type descriptions, command line menus, etc. [file 1471-2105-9-523-S1.zip › USeqUserGuides/JavaDoc.zip!/JavaDoc/util/xml/package-summary.html]

util.xml


|  |  |  |
| --- | --- | --- |
| **Overview** \| **Package** \| Class \| **Use** \| **Tree** \| **Deprecated** \| **Index** \| **Help** | |  |
| **PREV PACKAGE**   NEXT PACKAGE | **FRAMES**    **NO FRAMES**     **All Classes** |  |


---

## Package util.xml

| **Class Summary** | |
| --- | --- |
| **ParseXMLFile** | This class represents short example how to parse XML file, get XML nodes values and its values.   It implements method to save XML document to XML file too |

---


|  |  |  |
| --- | --- | --- |
| **Overview** \| **Package** \| Class \| **Use** \| **Tree** \| **Deprecated** \| **Index** \| **Help** | |  |
| **PREV PACKAGE**   NEXT PACKAGE | **FRAMES**    **NO FRAMES**     **All Classes** |  |


---
